# Effect of Stress on Viral–Bacterial Synergy in Bovine Respiratory Disease: Novel Mechanisms to Regulate Inflammation

**DOI:** 10.1002/cfg.474

**Published:** 2005-06

**Authors:** P. D. Hodgson, P. Aich, A. Manuja, K. Hokamp, F. M. Roche, F. S. L. Brinkman, A. Potter, L. A. Babiuk, P. J. Griebel

**Affiliations:** 1 Genome Prairie Suite 115, 3553-31 St., NW Calgary AB T2L 2K7 Canada; 2 Vaccine and Infectious Disease Organization 120 Veterinary Road University of Saskatchewan Saskatoon SK S7N 5E3 Canada; 3 Department of Molecular Biology and Biochemistry Simon Fraser University Burnaby BC V5A 1S6 Canada; 4 Central Institute for Research on Buffalo Sirsa Road Hisar Haryana 125001 India

## Abstract

The severity of bovine respiratory infections has been linked to a variety of factors, including environmental and nutritional changes, transportation, and social reorganization of weaned calves. Fatal respiratory infections, however, usually occur when a primary viral infection compromises host defences and enhances the severity of a secondary bacterial infection. This viral–bacterial synergy can occur by a number of different mechanisms and disease challenge models have been developed to analyse host responses during these respiratory infections. A primary bovine herpesvirus-1 (BHV-1) respiratory infection followed by a secondary challenge with *Mannheimia haemolytica* results in fatal bovine respiratory disease (BRD) and host responses to these two pathogens have been studied extensively. We used this disease model to demonstrate that stress significantly altered the viral–bacterial synergy resulting in fatal BRD. Functional genomic analysis revealed that BHV-1 infection enhanced toll-like receptors (TLR) expression and increased pro-inflammatory responses which contribute to the severity of a *Mannheimia haemolytica* infection. TLRs play a critical role in detecting bacterial infections and inducing pro-inflammatory responses. It is difficult to understand, however, how stress-induced corticosteroids could enhance this form of viral–bacterial synergy. Nuclear translocation of the glucocorticoid receptor activates cell signalling pathways which inhibit both TLR signalling and pro-inflammatory responses. The apparent conundrum between stress-induced corticosteroids and enhanced BRD susceptibility is discussed in terms of present data and previous investigations of stress and respiratory disease.


**Comparative and Functional Genomics**
*Comp Funct Genom* 2005; **6**: 244–250.
Published online in Wiley InterScience (www.interscience.wiley.com). **DOI:** 10.1002/cfg.474

**Conference Paper**

### Effect of stress on viral–bacterial synergy in bovine respiratory disease: novel mechanisms to regulate inflammation

P. D. Hodgson[1,2], P. Aich[2], A. Manuja[#], K. Hokamp[3], F. M. Roche[3], F. S. L. Brinkman[3], A. Potter[2], L. A. Babiuk[2] and P. J. Griebel[2]*

[1]*Genome Prairie, Suite 115, 3553-31 St., NW, Calgary, AB, Canada T2L 2K7*
[2]*Vaccine and Infectious Disease Organization, 120 Veterinary Road, University of Saskatchewan, Saskatoon, SK, Canada S7N 5E3*
[3]*Department of Molecular Biology and Biochemistry, Simon Fraser University, Burnaby, BC, Canada V5A 1S6*

**Correspondence to:*
*P. J. Griebel, Vaccine and Infectious Disease Organization, 120 Veterinary Road, University of Saskatchewan, Saskatoon, SK, Canada S7N 5E3.*
*E-mail: griebelp@sask.usask.ca*

*[#]Current Address: Central Institute for Research on Buffalo, Sirsa Road, Hisar, 125001 Haryana, India.*

Received: 17 February 2005
Accepted: 14 March 2005

### Abstract

**The severity of bovine respiratory infections has been linked to a variety of factors, including environmental and nutritional changes, transportation, and social reorganization of weaned calves. Fatal respiratory infections, however, usually occur when a primary viral infection compromises host defences and enhances the severity of a secondary bacterial infection. This viral–bacterial synergy can occur by a number of different mechanisms and disease challenge models have been developed to analyse host responses during these respiratory infections. A primary bovine herpesvirus-1 (BHV-1) respiratory infection followed by a secondary challenge with *Mannheimia haemolytica* results in fatal bovine respiratory disease (BRD) and host responses to these two pathogens have been studied extensively. We used this disease model to demonstrate that stress significantly altered the viral–bacterial synergy resulting in fatal BRD. Functional genomic analysis revealed that BHV-1 infection enhanced toll-like receptors (TLR) expression and increased pro-inflammatory responses which contribute to the severity of a *Mannheimia haemolytica* infection. TLRs play a critical role in detecting bacterial infections and inducing pro-inflammatory responses. It is difficult to understand, however, how stress-induced corticosteroids could enhance this form of viral–bacterial synergy. Nuclear translocation of the glucocorticoid receptor activates cell signalling pathways which inhibit both TLR signalling and pro-inflammatory responses. The apparent conundrum between stress-induced corticosteroids and enhanced BRD susceptibility is discussed in terms of present data and previous investigations of stress and respiratory disease. Copyright © 2005 John Wiley & Sons, Ltd.**

**Keywords: glucocorticoid receptor; IL-10; NFκB; pro-inflammatory cytokines; respiratory disease; stress; toll-like receptors (TLR)**

### Bovine respiratory disease (BRD) complex and viral–bacterial synergy

Increased risk of fatal bacterial respiratory infections following a primary viral infection has been observed in a wide range of species. This phenomenon is called viral–bacterial synergy and was first observed following human influenza epidemics, when a variety of secondary bacterial respiratory infections were associated with increased mortality [1,18,36]. Respiratory infections also remain a major economic and clinical problem in neonatal dairy calves, beef calves and other domestic ruminants, such as sheep and goats. These natural infections provide excellent disease models for investigating not only the interaction between viral

Copyright © 2005 John Wiley & Sons, Ltd.

and bacterial respiratory infections but also the role of stress in determining the incidence and severity of respiratory infections.

The aetiology and epidemiology of the BRD complex have been extensively reviewed elsewhere [4,9,20] and will only be discussed briefly. Fatal bovine respiratory infections are frequently characterized by a primary viral infection followed by a secondary bacterial infection. Viral pathogens implicated in this disease complex include bovine parainfluenza-3 virus, bovine respiratory syncytial virus, bovine viral diarrhea virus and bovine herpesvirurus-1 (BHV-1), which appears to be of primary importance. Bacterial pathogens implicated in acute and chronic BRD include *Pasteurella multocida, Haemophilus somnus* and *Mycoplasma* spp. The major bacterial pathogen involved in acute BRD appears to be *Mannheimia haemolytica* (M. haemolytica), a normal commensal microorganism present in the bovine upper respiratory tract. Thus, the concept has emerged that specific mechanisms must exist by which a primary BHV-1 infection can enhance bacterial colonization and virulence during a respiratory infection.

A variety of mechanisms have been proposed by which primary viral infections enhance bacterial colonization and invasion of the respiratory tract and alter host responses to bacterial infection. An experimental model of BRD, which consists of a primary BHV-1 respiratory challenge followed 4 days later by an aerosol challenge with *M. haemolytica*, has been used to consistently reproduce the clinical and pathological symptoms associated with fatal BRD infections. This model has been used extensively to identify the potential mechanisms underlying the viral–bacterial synergy that results in fatal BRD [4,32,33]. A variety of potential immune modulatory mechanisms have been identified that may play an important role in the viral–bacterial synergy observed following a primary BHV-1 infection. These mechanisms include altered alveolar macrophage function, altered polymorphonuclear cell function, decreased NK-cell activity and increased production of pro-inflammatory cytokines.

The increased production of pro-inflammatory cytokines that occurs during a primary BHV-1 is of particular interest in view of the pathology associated with an acute *M. haemolytica* respiratory infection [2]. Within hours of bacterial colonization of the lung there is a necrotizing inflammatory response that is characterized by the increased production of pro-inflammatory cytokines, such as IL-1, IL-8, and TNF$\alpha$ [31,42], and increased polymorphonuclear leukocyte recruitment to the lung [37]. The bacterial components that contribute to the activation of these inflammatory responses include capsule polysaccharide, lipopolysacchide (LPS), and leukotoxin [8,40]. It is now known that many of these bacterial components can directly stimulate pro-inflammatory responses in a wide variety of cells through interactions with toll-like receptors (TLRs) [5]. Thus, understanding the regulation of TLR signalling is critical for understanding the pathogenesis of bacterial diseases, such as *M. haemolytica* infection, which are characterized by the induction of profound inflammatory responses that cause destruction of lung tissue and sepsis.

### Role of stress in bovine respiratory disease

A link between stress and respiratory infections has been suggested by numerous studies in humans and animals. Epidemiological studies have linked a variety of psychological stressors with an increased incidence and severity of respiratory infections in humans [7,12,22]. A direct link between stress and increased severity of respiratory infections was suggested by increased levels of IL-6 in nasal secretions following influenza B challenge of individuals assessed with higher psychological stress [13]. Unfortunately, this study had no independent measurement, such as serum or salivary corticosteroid levels, to determine whether the perception of psychological stress correlated with a physiological effect.

Studies in mice have revealed a more complex interaction between psychological stress and respiratory infections. For example, restraint prior to or following intranasal challenge with influenza A virus resulted in reduced cellular infiltration in lungs and draining lymph nodes and reduced production of both Th1-type and Th2-type cytokines [24,34]. A glucocorticoid receptor antagonist was used in subsequent studies to confirm that the effects of stress on cell trafficking and cytokine production were mediated by elevated corticosterone [17]. Thus, in this experimental model the induction of elevated glucocorticoids by a psychological stressor was associated with a reduction in lung

Copyright © 2005 John Wiley & Sons, Ltd.

pathology and increased survival following a respiratory viral infection. In contrast, social reorganization was observed to increase both cellular infiltration in the lung and mortality following influenza challenge of mice, and this increased mortality was associated with a state of glucocorticoid insensitivity [35]. Thus, it was suggested that increased production of nerve growth factor (NGF) may have contributed to the development of steroid insensitivity. These studies demonstrated that different types of psychological stressors have markedly different effects on host responses to viral respiratory infections but did not investigate the interaction between stress and viral–bacterial synergy in respiratory disease.

The stressors associated with increased risk of BRD in weaned beef calves include restraint, social reorganization, transport, and nutritional changes [14]. There is contradictory evidence that the social reorganization associated with weaning (abrupt removal of calves from their dam) induces a stress response with elevated serum corticosteroids [15,28]. In contrast, short-term restraint of calves induces a rapid but transient increase in serum corticosteroids and transport for 3–4 h results in a 48–72 h increase in plasma corticosteroids, and transport has also been associated with increased mortality in young calves [38]. Thus, transportation has been a major focus of studies investigating the potential effect of stress on either immune function or respiratory disease in calves. Elevated serum corticosteroid levels immediately following transport have been associated with transient alterations in blood leukocyte function [6,19] as well as altered composition and function of bronchoalveolar lavage cells [26]. It has been more difficult, however, to demonstrate that transport and social reorganization have a significant impact on either morbidity or mortality due to BRD following a primary BHV-1 infection [19]. Furthermore, corticosteroid therapy, a potent suppressor of inflammatory responses, can be beneficial in the treatment of experimental *M. haemolytica* infection [29,39]. It should be noted, however, that corticosteroid therapy was effective in the absence of a primary viral infection. In conclusion, there is contradictory evidence that stress, especially as measured by elevated serum corticosteroids, enhances the viral–bacterial synergy that results in fatal BRD following infection by *M. haemolytica*.

### Interaction between stress and viral–bacterial synergy in BRD

The effects of different psychological stressors on respiratory viral infections in mice suggest that similar complex interactions probably occur among the psychological, physical and nutritional stressors associated with weaning and transport of calves. We initiated an analysis of the potential interactions among multiple stressors during BRD by using the disease model of a primary BHV-1 respiratory infection followed by an aerosol challenge with *M. haemolytica*. This disease model induces clinical signs of respiratory disease in all calves with a mortality rate of 30–70% [3]. A combination of social reorganization (abrupt separation of calf and dam) and transportation was compared to transportation alone immediately prior to BHV-1 infection, and the severity of a secondary *M. haemolytica* challenge was then monitored through clinical signs and mortality. Results showed that mortality due to BRD was twice as high (80%) in calves experiencing the combination of social reorganization and transport. Functional genomics analyses were then performed to identify possible mechanisms by which a primary viral infection might enhance the severity of a secondary bacterial infection. Gene expression patterns of the two groups of calves were further analysed to determine whether differences in serum corticosteroid levels might explain the marked difference in BRD mortality rates.

Microarray and quantitative RT-PCR analysis of blood mononuclear leukocytes confirmed significant differences in gene expression following BHV-1 infection when comparing the two groups of calves [25]. Both groups displayed conserved responses to BHV-1 infection, such as increased expression of interferon-induced genes (i.e. IFITM2; CA5B), pro-inflammatory genes (i.e. BIRC1) and toll-like receptors (i.e. TLR2 and TLR4). There were also many changes in gene expression associated with lipid metabolism, ion transport and cell growth. Many of these metabolic functions are regulated by glucocorticoids, which prompted us to analyse serum cortisol levels throughout the course of BHV-1 infection. As expected, both groups of calves had cortisolaemia immediately following transport and at the time of BHV-1 infection. However, only in the transport-alone group did serum cortisol levels remain significantly elevated at 24 h post-BHV-1 infection.

Copyright © 2005 John Wiley & Sons, Ltd. 

As expected from previous investigations [19,38], transport alone induced a cortisolaemia that persisted for 48 h, but when transport was combined with social reorganization then the cortisolaemia persisted for less than 24 h. Thus, there appeared to be an inverse correlation between the duration of cortisolaemia and mortality due to BRD. This is consistent with previous reports that elevated corticosteroids reduced the severity of influenza infections in mice [24,34] and treatment with glucocorticoids reduced the severity of *M. haemolytica* infections [29,39].

Glucocorticoids are potent inhibitors of inflammation by altering immune cell trafficking, effector cell activity, and inhibiting pro-inflammatory gene expression [10,16]. Elevated serum corticosteroid levels result in nuclear translocation of the glucocorticoid receptor and suppression of key transcriptional regulators of pro-inflammatory genes, such as NF$\kappa$B and AP-1 (Figure 1). Glucocorticoid

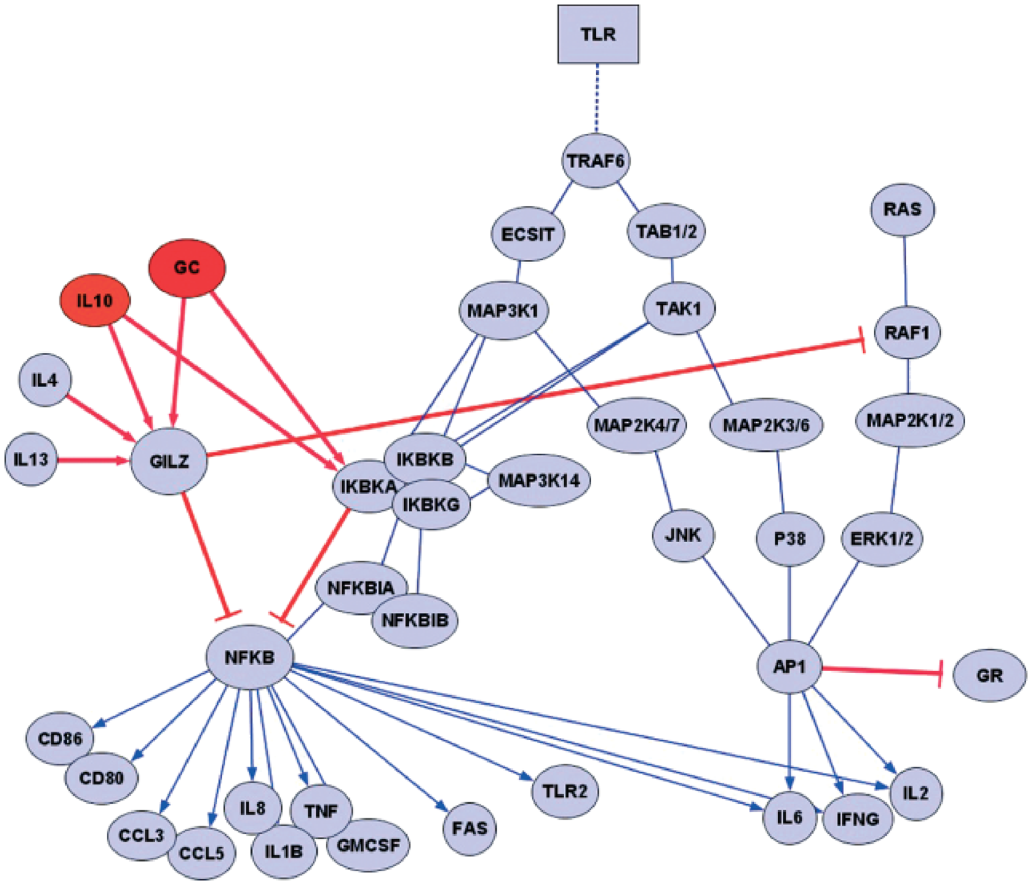

**Figure 1.** Potential mechanisms by which stress may inhibit downstream TLR signalling. Blue lines represent signalling pathways through which Toll-like receptors (TLRs) regulate the expression of pro-inflammatory cytokines. Red lines represent mechanisms by which molecules induced by stress, e.g. glucocorticoids (GC) and IL-10, or Th2 cytokines, e.g. IL-4 and IL-13, may inhibit pro-inflammatory gene expression through NF$\kappa$B and AP-1 transcription factors. Glucocorticoid receptor (GR) and IL-10 can inhibit NF$\kappa$B activity through upregulation of IKBKA, which sequesters NF$\kappa$B, thus preventing downstream gene transcription. In addition, GC and IL-10 can stimulate the production of glucocorticoid-induced leucine zipper protein (GILZ), which inhibits the functions of both NF$\kappa$B and AP-1. IL-4 and IL-13 inhibit NF$\kappa$B activity using a similar mechanism. The AP-1 complex can also negatively regulate the GC receptor (GR), thereby suggesting a negative feedback regulatory effect on the role of stress on TLR signalling. For simplicity, several nodes in this figure which represent multiple subunits are collapsed into a single node (e.g. p38, JNK, etc.). Standard HUGO names were used for genes when available. The following reports the alias names for these genes which are significantly different from the common gene name used: MAP3K1/MEKK1; MAP3K14/NIK; MAP2K4/MKK4; MAP2K7/MKK7; MAP2K3/MKK3; MAP3K6/MKK6; MAP2K1/MEK1; MAP2K2/MEK2; IKBKB/IKK$\beta$; IKBKG/IKK$\gamma$; IKBKA/IKK$\alpha$; NFKBIA/I$\kappa$B$\alpha$; NFKBIB/I$\kappa$B$\beta$

Copyright © 2005 John Wiley & Sons, Ltd. 

inhibition of NF$\kappa$B and AP-1 becomes of even greater interest when attempting to explain the potential mechanisms by which a primary viral infection can enhance the pathogenicity of a secondary bacterial respiratory infection. It was recently reported that human respiratory syncytial virus, which causes respiratory infections, induced increased TLR4 expression on blood monocytes [21]. In agreement with this observation, our functional genomic analyses revealed that BHV-1 infection increased the expression of both TLR2 and TLR4. TLR4 is required for LPS induction of pro-inflammatory cytokines, and LPS is an important molecule contributing to pulmonary responses to *M. haemolytica* infection [8,40]. Thus, increased TLR4 expression during BHV-1 infection potentially increases the host cell capacity to produce pro-inflammatory cytokines in response to *M. haemolytica* infection. Gene expression induced by TLRs, however, is mediated primarily through NF$\kappa$B and AP-1 and thus TLR-mediated responses can be modulated by the glucocorticoid receptor (Figure 1). Inhibition of TLR4 signalling by elevated glucocorticoids might be one possible explanation for the reduction in BRD mortality observed in the transport-alone group of calves which experienced a more sustained cortisolaemia.

Glucocorticoid inhibition of LPS-induced pro-inflammatory responses is not consistent with the observation that serum cortisol levels were close to baseline at the time of *M. haemolytica* challenge. There is, however, another potential mechanism by which corticosteroids may modulate pro-inflammatory responses induced by *M. haemolytica* infection. Another significant difference between the two groups of calves was an increased level of IL-10 expression in blood mononuclear cells isolated from the transport-alone calves [25]. IL-10 is a Th2-type cytokine with potent anti-inflammatory activity and this cytokine can inhibit induction of pro-inflammatory cytokines by LPS, which signals through TLR4 [23]. Glucocorticoids may influence the production of IL-10 indirectly by preventing dendritic cell (DC) maturation and immature DC play an important role in supporting $T_{regulatory}$ cell development [30]. $T_{regulatory}$ cells play an important role in regulating inflammatory responses through the production of several cytokines, including IL-10 and TGF$\beta$. Thus, increased IL-10 expression in blood mononuclear cells of the transport-alone group of calves may be the result of the sustained cortisolaemia which enhanced $T_{regulatory}$ cell development. Increased expression of IL-10, or other Th2-type cytokines such as IL-4 or IL-13, would then provide a mechanism (Figure 1) by which stress-induced glucocorticoids could modulate pro-inflammatory responses to LPS even after serum corticosteroids had returned to baseline values.

### Conclusions

Numerous epidemiological and experimental studies have provided substantial evidence that stress has a significant impact on the incidence and severity of respiratory infections. The interaction between stress and disease susceptibility is complex, with both the duration and nature of the stressor determining whether there is enhanced or decreased severity of the respiratory infection. BRD represents a disease complex with a variety of stressors implicated as potential contributing factors. We have used a bovine respiratory disease challenge model to begin defining molecular mechanisms by which stress may modulate the outcome of BRD infections. The model we chose, a secondary bacterial challenge with *M. haemolytica*, represents a respiratory infection in which the induction of an acute pro-inflammatory response and sepsis are major components of disease pathogenesis. Within this model, we identified two unique mechanisms by which stress-induced corticosteroids may modulate the viral–bacterial synergy which contributes to fatal bacterial infections. Corticosteroids may directly inhibit pro-inflammatory responses induced by increased TLR expression through inhibition of the transcriptional regulators NF$\kappa$B and AP-1. In addition, corticosteroids may indirectly regulate TLR-induced pro-inflammatory responses by supporting $T_{regulatory}$ cell development and increased IL-10 production. It should be noted, however, that for other secondary bacterial pathogens, such as *Haemophilus somnus*, pre-treatment with corticosteroid can exacerbate morbidity and mortality [11,27]. Thus, the effect of stress on the severity of BRD may depend as much on an interaction among multiple stressors as the specific pathogens involved in this respiratory disease complex.

Functional genomic analysis provided insight into the mechanisms by which stress-induced corticosteroids may modulate host responses to secondary bacterial respiratory infections. Another

Copyright © 2005 John Wiley & Sons, Ltd.

important question is the mechanism by which one specific stressor, such as social reorganization or weaning, was able to inhibit the cortisolaemia induced by transport. Weaning in combination with transport resulted in a truncated cortisolaemia, a failure to upregulate expression of IL-10 and a two-fold increase in BRD mortality. These observations implicated corticosteroids as a critical factor in determining the outcome of BRD but suggest that other factors may also contribute to the interaction between stress and host responses. NGF has been implicated as one factor that may increase the severity of respiratory infections and the source of NGF was the salivary gland [37]. Further functional genomic analysis of the interaction between stress and disease resistance should, therefore, include a variety of tissues that may produce important immunoregulatory molecules. The interaction between stress and the immune system represents only one aspect of the complex interaction between stress and the physiology and metabolism of the whole organism.

### Acknowledgements

This manuscript is published by permission of the Director of VIDO as Journal Series No. 398 and the work was supported by funding provided by Inimex Pharmaceuticals (Vancouver, BC) and Genome Prairie/Genome BC, in part through Genome Canada. K.H. and F.S.L.B. are a Micheal Smith Foundation for Health Research Trainee and Scholar, respectively, and L.A.B. is a holder of the Canada Research Chair in Vaccinology.

### References

1. Abrahams A, Hallows N, French H. 1919. A further investigation into influenzo-pneumococcal and influenzo-streptococcal septicaemia. *Lancet* **1**: 1–11.
2. Ackermann MR, Brogden KA. 2000. Response of the ruminant respiratory tract to *Mannheimia* (*Pasteurella*) *haemolytica*. *Microb Infect* **2**: 1079–1088.
3. Babiuk LA, Lawman MJ, Gifford GA. 1987. Use of recombinant bovine $\alpha$1-interferon in reducing respiratory disease induced by bovine herpesvirus type 1. *Antimicrob Agents Chemother* **31**: 752–757.
4. Babiuk LA, van Drunen Little-van den Hurk S, Tikoo SK. 1996. Immunology of bovine herpesvirus 1 infection. *Vet Microbiol* **53**: 31–42.
5. Beutler B. 2004. Inferences, questions, and possibilities in toll-like receptor signaling. *Nature* **430**: 257–263.
6. Blecha FS, Boyle L, Riley JG. 1984. Shipping suppresses lymphocyte blastogenic responses in Angus and Brahman X Angus feeder calves. *J Anim Sci* **59**: 576–583.
7. Boyce WT, Jensen EW, Caseel JC, Collier AM, Smith AH. 1977. Influence of life events and family routines in childhood respiratory tract illness. *Pediatrics* **60**: 609–615.
8. Brogden KA, Ackermann MR, Debey MD. 1995. Purified lipoopolysaccharide-associated protein from *Pasteurella haemolytica* induces pulmonary inflammation in calves and sheep. *Infect Immun* **63**: 3595–99.
9. Brogden KA, Lehmkuhl HD, Cutlip RC. 1998. *Pasteurella haemolytica* complicated respiratory infections in sheep and goats. *Vet Res* **29**: 233–254.
10. Buckbinder L, Robinson RP. 2002. The glucocorticoid receptor: molecular mechanism and new therapeutic opportunities. *Curr Drug Targets Inflamm Allergy* **1**: 127–136.
11. Chiang YW, Roth JA, Andrews JJ. 1990. Influence of recombinant bovine interferon-$\gamma$ and dexamethasone on pneumonia attributable to *Haemophilus somnus* in calves. *Am J Vet Res* **51**: 759–762.
12. Clover RD, Abell T, Becker LA, Crawford S, Ramsey CN. 1989. Family functioning and stress as predictors of influenza B infection. *J Fam Pract* **28**: 536–539.
13. Cohen S, Doyle WJ, Skoner DP. 1999. Psychological stress, cytokine production, and severity of upper respiratory illness. *Psychosom Med* **61**: 175–180.
14. Cole NA. 1996. Review of bovine respiratory disease: nutrition and disease interactions. In *Review of Bovine Respiratory Disease*, Smith R (ed.). Schering Plough Animal Health, Veterinary Learning Systems: Trenton, NJ; 57–74.
15. Coppo JA, Mussart NB, Revidatti MA, Capellari A. 2003. Absence of biochemically demonstrable stress in early weaned half-bred Zebu calves. *Cien Inv Agr* **30**: 97–105.
16. de Bosscher K, vanden Berghe W, Haegeman G. 2003. The interplay between the glucocorticoid receptor and nuclear factor-$\kappa$B or activator protein-1: molecular mechanisms for gene expression. *Endocr Rev* **24**: 488–522.
17. Dobbs CM, Feng N, Beck FM, Sheridan JF. 1996. Neuroendocrine regulation of cytokine production during experimental influenza viral infection. *J Immunol* **157**: 1870–1877.
18. Farr W. 1885. In *Vital Statistics: A Memorial Volume of Selections from Reports and Writings of William Farr*, Humphrys NA (ed.). Office of the Sanitary Institute: London; 330.
19. Filion LG, Willson PJ, Bielefeldt-Ohmann H, Babiuk LA, Thompson RG. 1984. The possible role of stress in induction of pneumonic pasteurellosis. *Can J Comp Med* **48**: 268–274.
20. Frank GH, Briggs RE, Loan RW, Purdy CW, Zehr ES. 1996. Respiratory disease and mucosal colonization by *Pasteurella haemolytica* in transported cattle. *Am J Vet Res* **57**: 1317–1320.
21. Gagro A, Tominac M, Krsulovic-Hresic V, *et al*. 2004. Increased toll-like receptor 4 expression in infants with respiratory syncytial virus bronchiolitis. *Clin Exp Immunol* **135**: 267–272.
22. Graham NMH, Douglas RB, Ryan P. 1986. Stress and acute respiratory infection. *Am J Epidemiol* **124**: 389–401.
23. Grutz G. 2005. New insights into the molecular mechanism of interleukin-10-mediated immunosuppression. *J Leuk Biol* **77**: 3–15.
24. Hermann G, Trovar CA, Beck FM, Allen C, Sheridan JF. 1993. Restraint stress differentially affects the pathogenesis of an experimental influenza viral infection in three inbred strains of mice. *J Neuroimmunol* **47**: 83–92.

Copyright © 2005 John Wiley & Sons, Ltd. 

25. Hodgson PD, Aich P, Manuja A, *et al.* 2005. Effect of stress on viral–bacterial synergy in bovine respiratory disease. Plant and Animal Genomics XIII Conference, San Diego, CA; 698.
26. Ishizaki H, Hanafusa Y, Kariya Y. 2005. Influence of truck-transportation on the function of broncho-alveolar lavage fluid cells in cattle. *Vet Immunol Immunopathol* (in press).
27. Lefcourt AM, Elsasser TH. 1995. Adrenal responses of Angus X Hereford cattle to the stress of weaning. *J Anim Sci* **73**: 2669–2676.
28. Jackson JA, Andrews JJ, Hargis JW. 1987. Experimental *Haemophilus somnus* pneumonia in calves. *Vet Pathol* **24**: 129–134.
29. Malazdrewich C, Thumbikat P, Maheswaran SK. 2004. Protective effect of dexamethasone in experimental bovine pneumonic mannheimiosis. *Microbiol Pathogen* **36**: 227–236.
30. Matyszak MK, Citterio S, Rescigno M, Ricciardi-Castagnoli P. 2000. Differential effects of corticosteroids during different stages of dendritic cell maturation. *Eur J Immunol* **30**: 1233–1242.
31. Morsey MA, van Kessel AG, Mori Y, *et al.* 1999. Cytokine profiles following interaction between bovine alveolar macrophages and *Pasteurella haemoloytica*. *Microb Pathog* **26**: 325–331.
32. Ohmann HB, Babiuk LA. 1985. Viral–bacterial pneumonia in calves: effect of bovine herpesvirus-1 on immunologic functions. *J Infect Dis* **151**: 937–947.
33. Ohmann HB, Babiuk LA, Harland R. 1991. Cytokine synergy with viral cytopathic effects and bacterial products during the pathogenesis of respiratory tract infection. *Clin Immunol Immunopathol* **60**: 153–170.
34. Sheridan JF, Feng N, Bonneau RH, *et al.* 1991. Restraint differentially affects anti-viral cellular and humoral immune responses in mice. *J Neuroimmunol* **31**: 245–253.
35. Sheridan JF, Stark JL, Avitsur R, Padgett DA. 2000. Social disruption, immunity, and susceptibility to viral infection. Role of glucocorticoid insensitivity and NGF. *Ann NY Acad Sci* **917**: 894–905.
36. Simonsen L, Fukuda K, Schonberger LB, Cox NJ. 2000. The impact of influenza epidemics on hospitalizations. *J Infect Dis* **181**: 831–837.
37. Slocombe RF, Malark J, Ingersoll R, Derksen FJ, Robinson NE. 1985. Importance of neutrophils in the pathogenesis of acute pneumonic pasteurellosis in calves. *Am J Vet Res* **46**: 2253–2258.
38. Stephens DB. 1980. Stress and its measurement in domestic animals: a review of behavioural and physiological studies under field and laboratory situations. *Adv Vet Sci Comp Med* **24**: 179–210.
39. Sustronck B, Deprez P, van Loon G, Coghe J, Muylle E. 1997. Efficacy of the combination sodium ceftiofur–flumethasone in the treatment of experimental *Pasteurella haemolytica* bronchopneumonia in calves. *Zent Vet A* **44**: 179–187.
40. Whiteley LO, Mahaswaran SK, Weiss DJ, Ames TR. 1991. Morphological and morphometrical analysis of the acute response of the bovine alveolar wall to *Pasteurella haemolytica* A1-derived endotoxin and leucotoxin. *J Comp Pathol* **104**: 23–32.
41. Yates WDG. 1982. A review of infectious bovine rhinotracheitis, shipping fever pneumonia and viral–bacterial synergism in respiratory disease in cattle. *J Comp Med* **46**: 225–263.
42. Yoo HS, Maheswaran SK, Srinand S, Ames TR, Suresh M. 1995. Increased tumour necrosis factor-$\alpha$ and interleukin-1$\beta$ expression in the lungs of calves with experimental pneumonic pasteurellosis. *Vet Immunol Immunopathol* **49**: 15–28.

Copyright © 2005 John Wiley & Sons, Ltd. 
